# Investigations into Structural Behavior of Concrete-Filled RHS Columns with Unequal Flange Thickness under Compressive Load

**DOI:** 10.3390/ma13235463

**Published:** 2020-11-30

**Authors:** Guangyuan Fu, Gongyi Fu, Siping Li, Jian Yang, Feiliang Wang

**Affiliations:** 1 State Key Laboratory of Ocean Engineering, Shanghai Jiao Tong University, Shanghai 200240, China; sjtu468800@sjtu.edu.cn (G.F.); gyfu@sjtu.edu.cn (G.F.); lisp@mail.tsinghua.edu.cn (S.L.); j.yang.1@sjtu.edu.cn (J.Y.); 2 Shanghai Key Laboratory for Digital Maintenance of Buildings and Infrastructure, School of Naval Architecture, Ocean and Civil Engineering, Shanghai Jiao Tong University, Shanghai 200240, China; 3 School of Civil Engineering, University of Birmingham, Birmingham B15 2TT, UK

**Keywords:** concrete-filled rectangular hollow section, eccentric compressed, experimental study, numerical simulation, load-bearing capacity

## Abstract

Previous studies have shown that components with an unequal-walled concrete-filled rectangular hollow section (CFRHS) can achieve a greater resistance under bending than those with equal-walled CFRHS. However, the study on the compressive behavior of the CFRHS column is limited. Therefore, this paper investigates the performance of compressed CFRHS columns with unequal flange thickness, based on experimental and numerical approaches. In the test, the effects of slenderness and eccentricity on the compressive capacity of the CFRHS columns with unequal shell thickness are discussed. Numerical models based on the finite element method are established, to evaluate the resistance and failure pattern of each specimen in the test. Parametric studies are carried out based on the validated model, to investigate the effect of eccentricity, wall thickness, and steel and concrete material properties on the load-bearing capacity of the compressed CFRHS column. In addition, the analytical expressions of the resistance of CFRHS columns with unequal wall thickness are derived, and the prediction values are validated through comparing with the test results. It is found that eccentric compressed columns with unequal-walled CFRHS have a similar load-bearing capacity and better ductility when compared with the equal-walled CFRHS.

## 1. Introduction

Concrete-filled steel hollow tubes have been widely applied in columns or piers due to their excellent properties, such as high strength, high ductility, and construction convenience. The composite columns have been investigated in previous research studies, covering varying loading scenarios, such as bending [1,2,3], compression [4,5,6,7,8,9], seismic [10], and different configurations, i.e., circular [11,12], square [13], and octagonal [14] sections. Lu and Kennedy [1] conducted a series of flexural tests on concrete-filled steel hollow sections, to assess the structural behavior of these composite components. The tests showed that the ultimate flexural strength and stiffness of the concrete-filled sections is increased by about 10~30% than that only with steel sections. Jiang et al. [2] carried out a series of bending tests on thin-walled concrete-filled steel tubes (CFST) and found that both American and European standards conservatively predict the bending strength of the tested specimens. To extend the experimental research, Moon et al. [3] proposed an analytical model to predict the flexural behavior of CFST based on numerical simulations. They also carried out a series of parametric studies to investigate the effects of width-to-thickness (w/t) ratio and materials properties based on the validated model. Han [4] found that the constraining factor has a significant impact on both compressive resistance and ductility of the CFST stub columns. To enhance the axial compression performance of CFST columns, Lyu et al. [5] used reinforcement stiffeners, to prevent buckling on the steel outer and to enhance the restraint effect on the concrete. Liu et al. [6] investigated the effect of high-strength steel fiber reinforced concrete on the axial compression behavior of rectangular-sectional CFST columns. Cha et al. [7] found the diaphragm had significant effects on the compressive strength and toughness of the CFST columns. Hasan et al. [8] presented the mechanical performance of CFST columns strengthened by steel reinforcing bars (RBs) under axial compression. Zhang et al. [9] investigated the compressive performance of steel-reinforced concrete-filled circular steel tubular (SRCFT) stub columns and proposed a novel simplified formula to predict the ultimate bearing capacity of SRCFT stub columns. Moreover, the experimental results indicate that cyclic loading has limited influence on the stiffness or strength of CFST beam–column joints [10]. Johansson and Gyllotoft [11] studied the circular steel–concrete composite columns and observed that the mechanical behavior of the column was considerably influenced by the loading condition. The bond strength was found to have no influence on the behavior of the column when the steel and concrete sections were loaded simultaneously. On the contrary, for the column with the load applied only to the concrete section, the bond strength highly affected the confinement effect of the concrete and the mechanical behavior of the column. Then, Lu et al. [12] proposed a strut–tie model to predict the load transfer mechanism of the circular composite member subjected to pure bending. Lu et al. [13] investigated the bending moment-to-curvature relationship for eccentrically compressed square CFST columns and presented an analytical method for evaluating the ultimate strength of the column based on the collapse theory.

**Figure 1:**
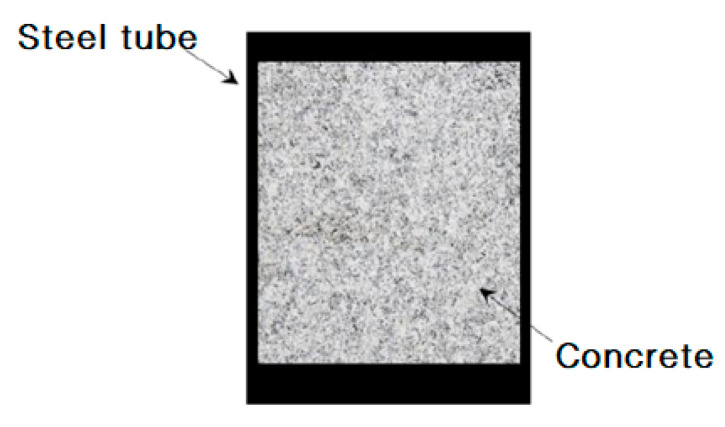
Cross-section of concrete-filled rectangular hollow section (CFRHS) with an unequal wall thickness.

It was indicated that about 3/4 of the concrete was out-of-work in the tension zone of the concrete-filled rectangular hollow section (CFRHS) steel-tube bending members with equal wall thickness when the failure occurred, and the contribution of the steel web to the bending bearing capacity was relatively small [15]. Then, Lu and Li [15] initially proposed a CFRHS steel tube with an unequal wall thickness, as shown in Figure 1. They found form laboratory tests that the unequal-walled flexural members have better ductility and load-bearing capacity under flexural than that with equal-walled sections. Zhang and Yu et al. [16,17] investigated the elastic–plastic stage of asymmetric rectangular CFST beams with unequal wall thickness subjected to pure bending. The generalized bending formula and the generalized eccentric compression formula were deduced, and the equation of the neutral axis was provided. It was found that, for an asymmetric cross-section, the neutral axis will move and rotate nonlinearly with the plastic development of the section. 

The unequal-walled CFRHS can also introduce a benefit when adopted as an eccentric compressed member. When a unequal-walled CFRHS column is subjected to eccentric compression, the eccentricity of the section can be reduced, as the centroid will move to the side with greater wall thickness, hence improving the load-bearing capacity of the column. Therefore, unequal-walled CFRHS components are more suitable to be constructed as the side or corner columns than those with equal-walled CFRHS. To date, the structural behavior of the unequal-walled CFRHS column has not been fully studied. The influences of its eccentricity, material strength, sectional thickness, etc., on the load-bearing performance of the column still need to be revealed. Therefore, this paper investigates the load-bearing capacity of CFRHS column with unequal flange thickness under compression. Based on the test and simulation results, the analytical models of the column resistance are further proposed. This paper aims to provide a useful prediction method for engineers in the design of CFRHS columns under compression.

## 2. Experimental Studies

### 2.1. Test Program

In the test, 4 axially loaded and 8 eccentrically loaded CFRHS columns were prepared according to the following design variables: (1) slenderness (λ) is 27.7 or 37.0 (λ = 2$\sqrt{3}L/h$); (2) eccentricity (e) is 0, 20, or 40; (3) ratio between top and the bottom flange thickness (*t*_2_/*t*_1_) is 1.0 or 1.65. The rectangular steel tube was welded by the top and bottom endplates, with a thickness of 20 mm.

Geometric parameters for each test specimen are listed in Table 1. In the table, the first letters “S” and “D” for each specimen represent bi-axially symmetrical and uni-axially symmetrical sections, respectively. The yield strengths of the steel top flange (*f_y_*_1_), bottom flange (*f_y_*_2_), and web (*f_y_*_w_) were obtained via coupon tests. The compressive strength of the concrete (*f_cu_*) was achieved by cubic tests with a cube length of 100 mm.

**Figure 2:**
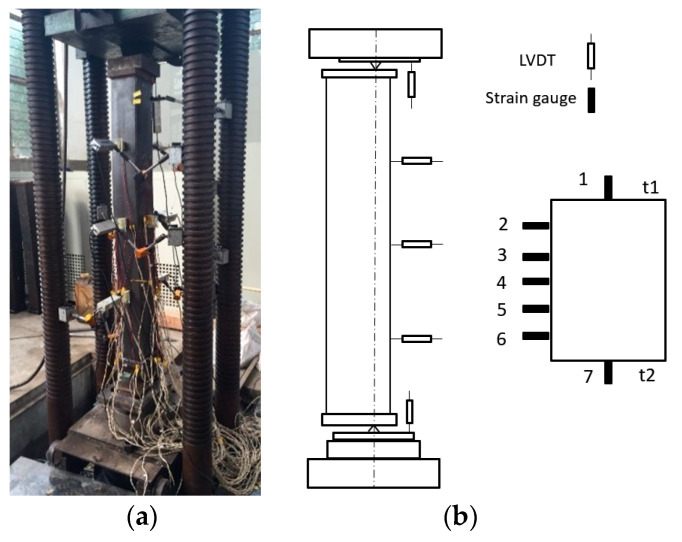
Test setup: (**a**) photograph of the test setup and (**b**) layout of the LVDT and strain gauges.

The test was carried out on a 200 t MTS hydraulic servo loading system, in the Structural Laboratory of Shanghai Jiao Tong University. The pin support condition was offered by using knife-edge hinges on both ends of each column specimen, and the schematic diagram of the loading device and the layout of Linear Variable Differential Transformers (LVDT) and strain gauges are shown in Figure 2.

### 2.2. Test Results

**Figure 3:**
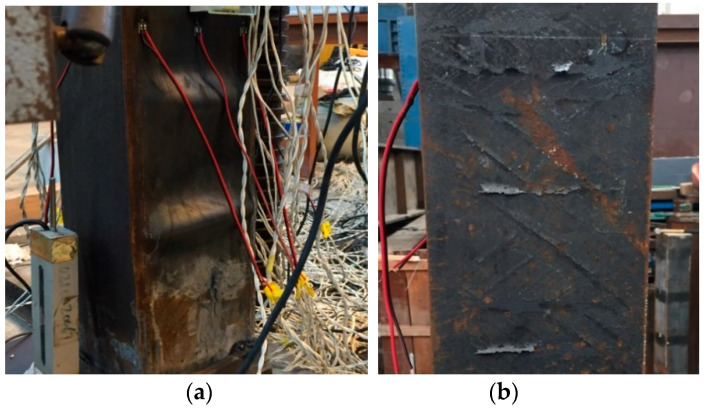

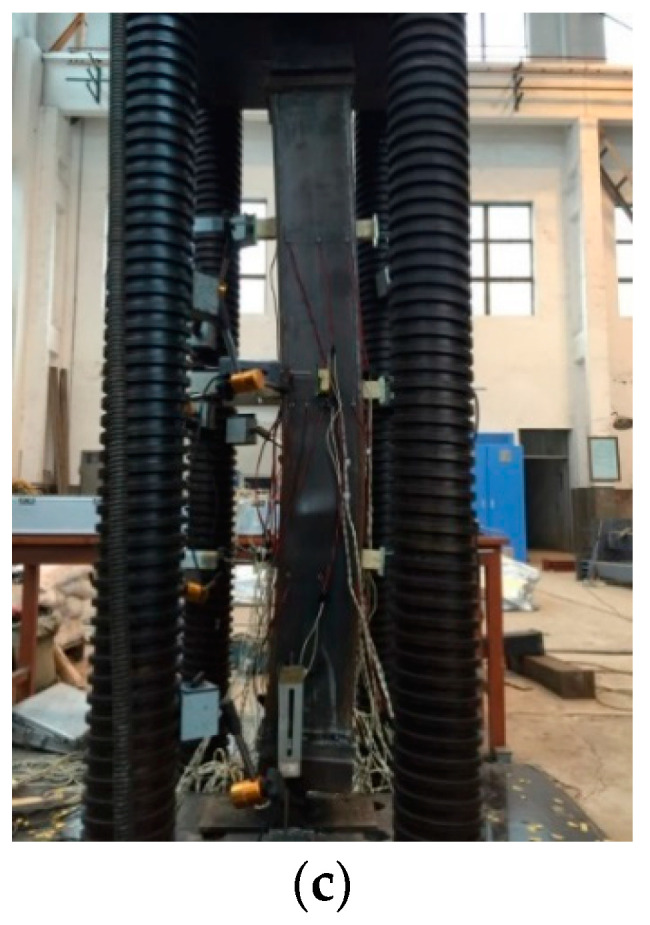
Failure pattern: (**a**) local buckling on the axially loaded column, (**b**) slip line on the eccentrically loaded column, and (**c**) failure mode of the eccentric compressed column.

For axially compressed specimens, the loading response of the specimen can be divided into three stages, i.e., elastic stage, elastic-to-plastic stage, and failure stage. In the initial stage, the behavior of steel tube and concrete is elastic, and the load-to-deflection curve ascends linearly. When the load increases to 70–80% of the ultimate capacity, the steel tube enters into the yield stage, and local buckling can be observed on the steel tube. As the compressive load keeps increasing, the buckle of the tube becomes more severe until the failure reached. The failure mode of the axial compression member is the local buckling of the tube on the top side (see Figure 3a). This is because the concrete at the bottom of the tube is better vibrated than the top concrete, which makes the bottom side of the column stiffer than the top side. Therefore, the concrete near the top side reaches the compressive strength earlier than the bottom side and causes a local buckling on the section. 

For the eccentrically loaded specimens, in the initial stage, the axial deformation is limited and the column is in the elastic state. When the load reaches 70–80% of the ultimate capacity, the flange and the sidewall gradually reach the yield strength, and 45° angle slip lines are observed on the steel tube, as shown in Figure 3b; the failure mode of the column is shown in Figure 3c. It can be seen that the largest deflection occurs close to the middle-height section of the column, and a local bucking also can be observed on the section.

**Figure 4:**
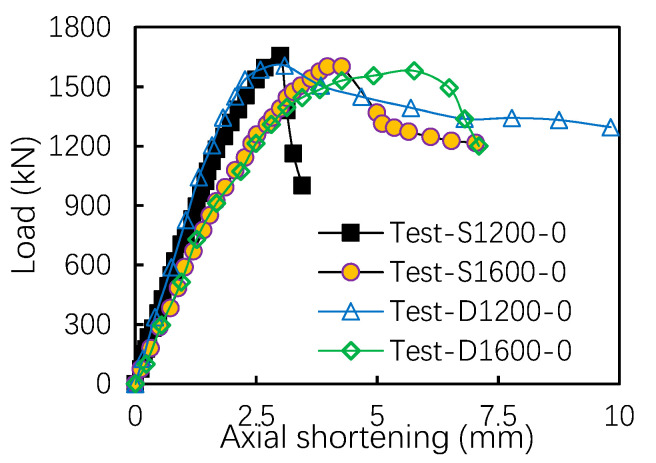
Load–shortening curves for axially loaded specimens.

For the axially compressed specimens, the axial stiffness of the uni-axially symmetrical members (test pieces D1200-0 and D1600-0) and the bi-axially symmetrical members (test pieces S1200-0 and S1600-0) are similar (see Figure 4). From the comparison, it is found that the load–displacement curve of uni-axially symmetrical section members is more ductile than that of bi-axially symmetrical members.

**Figure 5:**
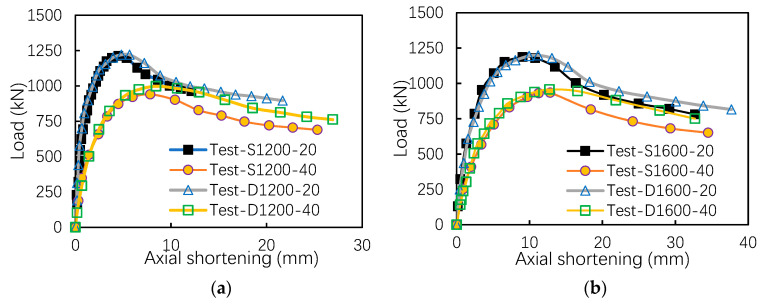
Load–shortening curves for eccentrically loaded specimens: (**a**) L = 1200 mm and (**b**) L = 1600 mm.

It is shown in Figure 5 that, as the eccentricity increases, both initial stiffness of the load–deflection curve and the ultimate load-bearing capacity decrease. The ductility for the eccentrically compressed members is found to be better than that of axially compressed members, especially in the failure stage. Comparing the load–deflection curves of uni-axially symmetrical members and bi-axially symmetrical members, it can be observed that, under the same eccentricity, the ultimate capacity of the columns with different flange thickness is slightly higher than that of columns with equal flange thickness. The reason is that the physical centroid of the section is close to the thicker flange for the uni-axially symmetrical specimens; thus, the actual eccentricity of the column is reduced. 

**Figure 6:**
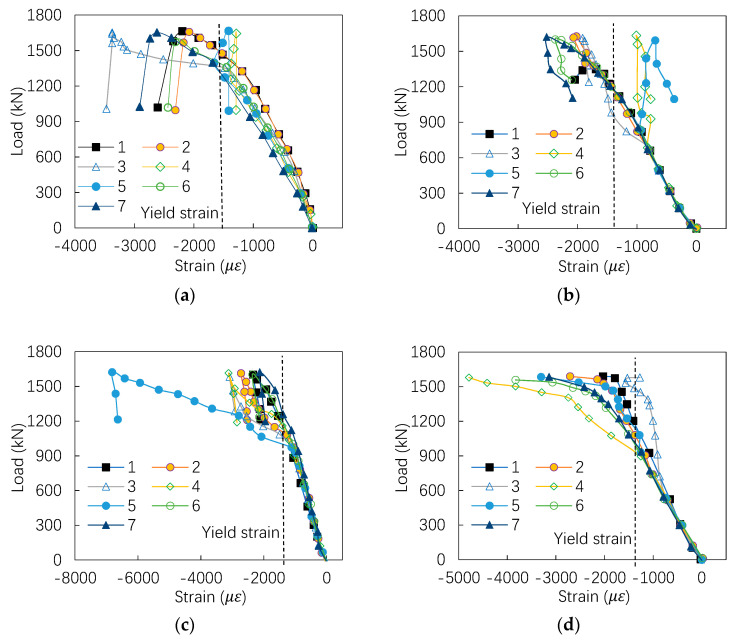

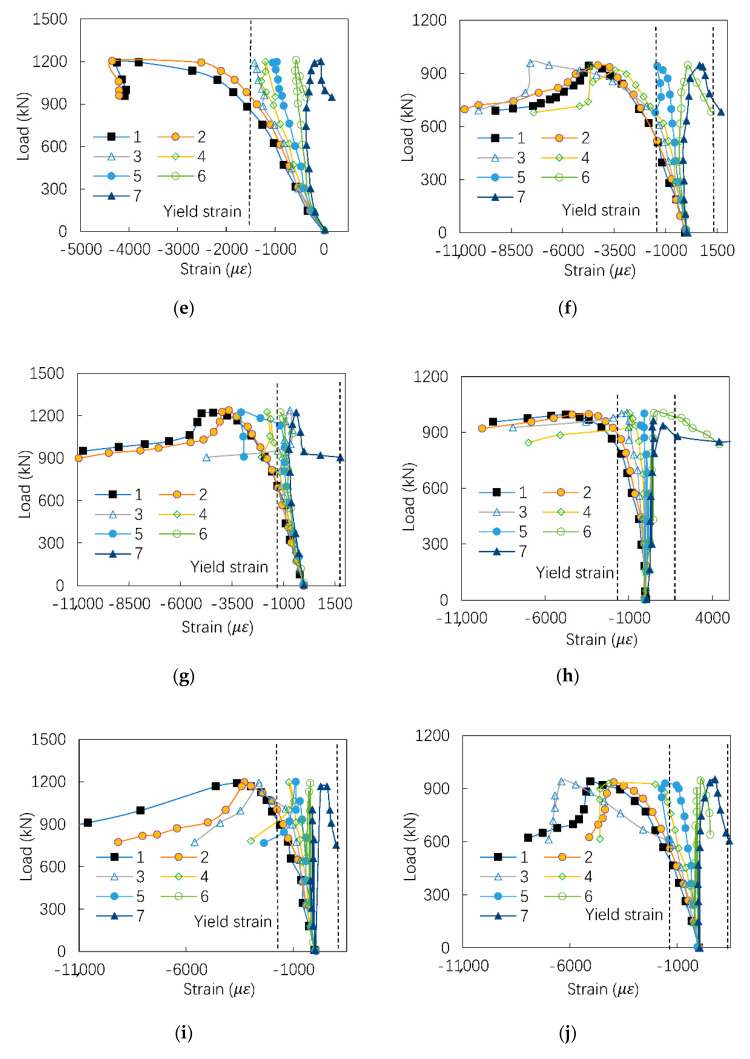

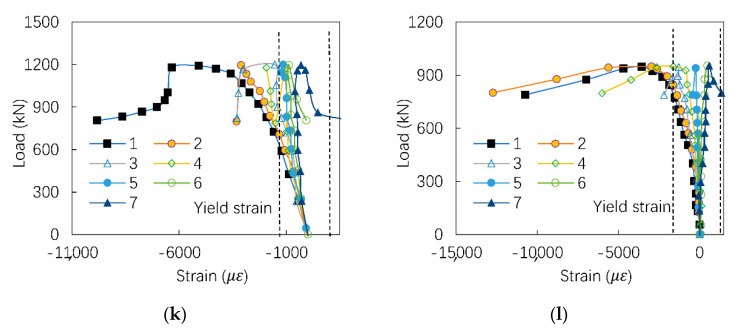
Load-versus-strain curves: (**a**) S1200-0, (**b**) D1200-0, (**c**) S1600-0, (**d**) D1600-0, (**e**) S1200-20, (**f**) S1200-40, (**g**) D1200-20, (**h**) D1200-40, (**i**) S1600-20, (**j**) S1600-40, (**k**) D1600-20, and (**l**) D1600-40.

The load-versus-longitudinal-strain curves are shown in Figure 6, where the strain is obtained from the strain gauge mounted on the middle section of the specimen. The numbers 1~7 in the figure represent the number of the strain gauge, and the position of the strain gauge is shown in Figure 2. It can be seen that, for the axially compressed members, all the strains on the mid-height section reach the yield strain with increasing loading. For the eccentrically loaded members, only part of the strain gauges reaches the yield strain when the column reaches the ultimate load-bearing capacity. Meanwhile, greater tensile strain is observed on the flange far away from the loading point than the near flange. It is visible that the columns with equal wall thickness have a sudden drop when the peak load achieved, while the columns with unequal wall thickness descend in slowly. This leads to the conclusion that the columns with unequal wall thickness have greater load-bearing capacity when compared with the columns with equal wall thickness, after approaching the peak load.

## 3. Numerical Analysis

### 3.1. Modeling Process

Finite element (FE) analysis program ABAQUS [18] was employed for nonlinear numerical modeling. The method proved to be a useful tool in simulation composite columns under compression. Tao et al. [19] develop refined FE models to simulate CFST stub columns under axial compression. The model was established based on the concrete damaged plasticity (CDP) material model, while the dilation angle used in the model was calibrated against test data and a new concrete strain hardening/softening function was developed. Zhang et al. [20] used a fiber beam-column model to simulate the nonlinear behavior of concrete-filled round-ended (CFRE) steel tubes. It was shown that the proposed fiber model was capable of simulating the statically and pseudo-statically loaded CFRE steel tubes with high accuracy via comparison to experimental results. Meanwhile, the FE analysis of CFRE steel tubes under bending, cyclic loading and axial loading indicated that the numerical value is lower than test value due to the strength deterioration of steel tubes in the CFRE beam–column members. Alatshan et al. [21] developed an explicit FE model to investigate the failure modes, axial load-to-strain relationship, stress distributions, and bond strength of the CFST column components. Shin et al. [22] presented a numerical study to investigate the performance of a plate-reinforced moment-resisting connection, using a large concrete-filled tubular (CFT) column subjected to blast. The modeling methodology was validated based on the existing experimental study and verified by using an alternate FE code.

**Figure 7:**
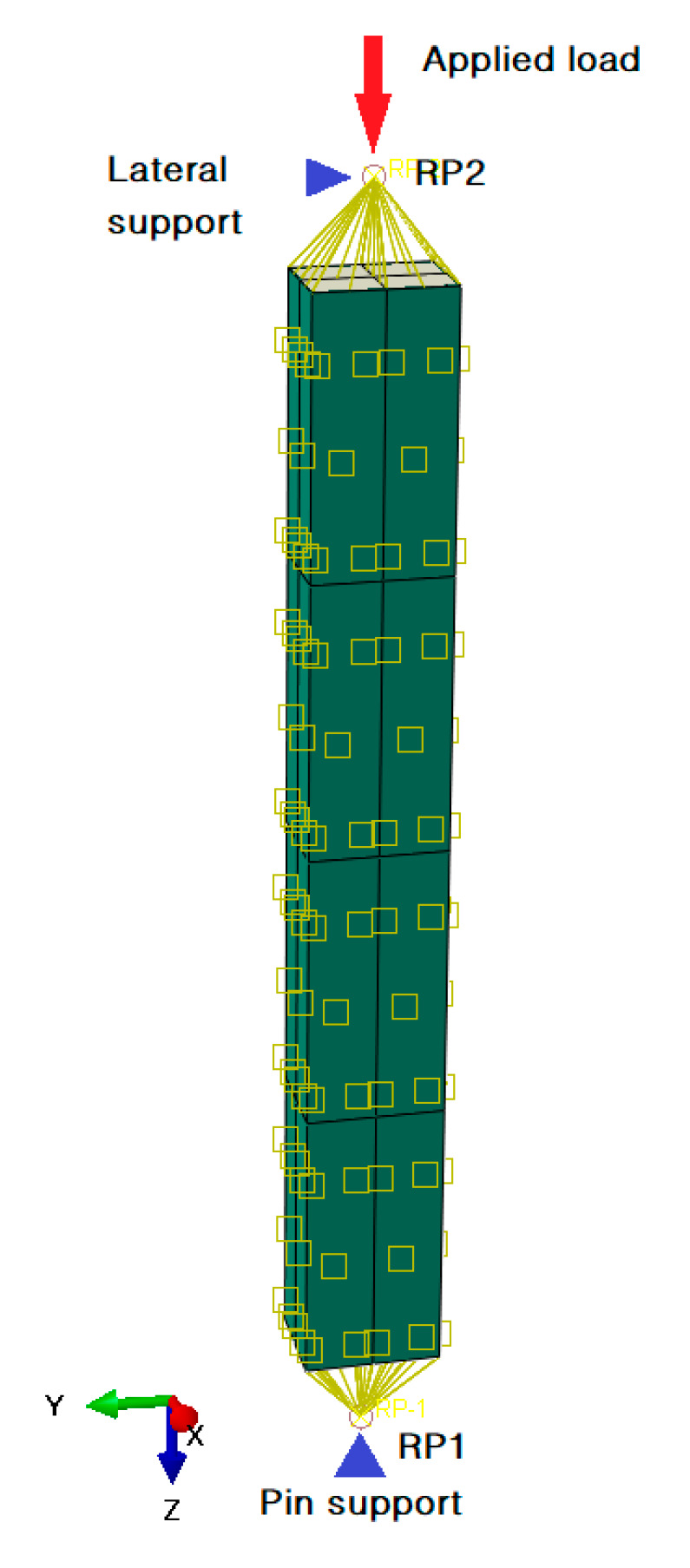
Boundary conditions in the model.

In the model, the steel tube was represented by four-node nonlinear shell element S4R, and the concrete was simulated by eight-node reduced integral element C3D8R. A surface-to-surface contact was defined between concrete (slave surface) and steel tube (master surface). Penalty contact was assumed in the normal direction of the contact to prevent penetration, and friction contact was adopted in the tangential direction with a coefficient of 0.6 [23]. The lower end of the column is restrained by pin support, and the upper end is loaded via a reference point, and the reference point and the section are coupled as shown in Figure 7. 

**Figure 8:**
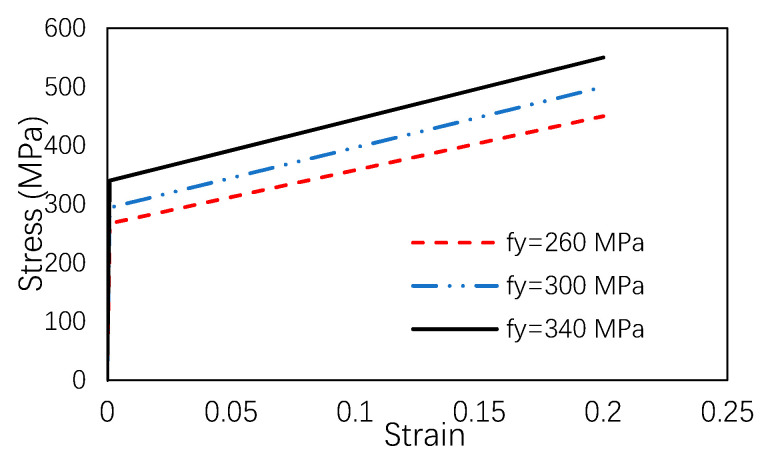
Stress–strain curve of the steel.

The mesh size in the model was 20 mm. The friction factor and mesh pattern were decided based on the sensibility analysis. In this paper, the Mises yield criterion was used to describe the failure of the column. Bilinear stress–strain curves were adopted in the constitutive model of steel according to the coupon test, as shown in Figure 8. The elastic modulus of the steel is 203 GPa, and the Poisson’s ratio is 0.3.

Concrete material in the model was simulated by CDP in ABAQUS, and the strain–stress curve is according to the equation proposed by Saenz [24]. In the CDP model, it assumes tensile cracking and compressive crushing as a failure mechanism for concrete. Input parameters such as dilation angle, eccentricity, biaxial loading ratio, the coefficient “K”, and viscosity parameter were 20, 0.1, 1.16, 0.667, and 0.001, respectively.

(1) $$f_{c}=\frac{E_{c}\varepsilon_{c}}{1+\left(R+R_{E}-2\right)\frac{\varepsilon_{c}}{\varepsilon_{c}^{\prime }}-\left(2R-1\right){\left(\frac{\varepsilon_{c}}{\varepsilon_{c}^{\prime }}\right)}^{2}+R{\left(\frac{\varepsilon_{c}}{\varepsilon_{c}^{\prime }}\right)}^{3}}$$

(2) $$R=\frac{R_{E}\left(R_{\sigma }-1\right)}{{\left(R_{\varepsilon }-1\right)}^{2}}-\frac{1}{R_{\varepsilon }}$$

(3) $$R_{E}=\frac{E_{c}\varepsilon_{c}^{\prime }}{f_{c}^{\prime }}$$

where *E_c_* is elasticity mule, *E_c_* = $\sqrt{4700f_{c}^{\prime }}$. according to ACI 318-14; $\varepsilon_{c}^{\prime }$. is the strain when compressive strength is achieved, $\varepsilon_{c}^{\prime }=0.003$ according to ACI 318-14.

**Figure 9:**
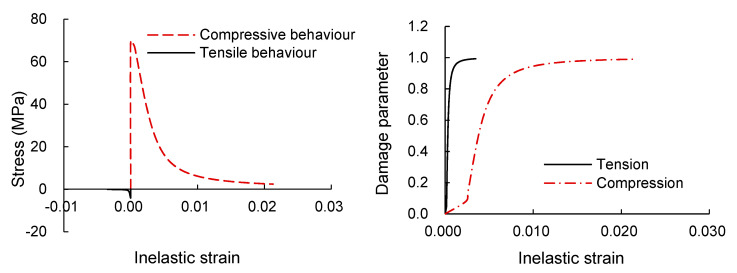
CDP properties of the concrete.

The detailed behavior of the material properties of concrete is shown in Figure 9.

### 3.2. Model Validation

**Figure 10:**
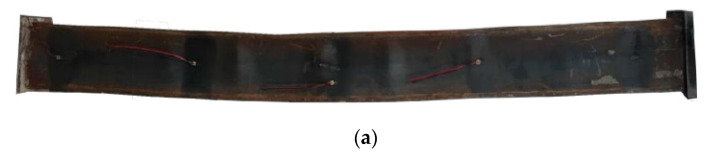

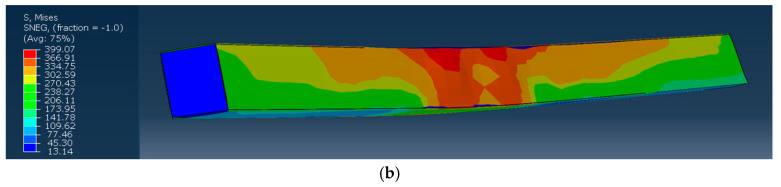
Comparison of the failure mode (S1600-0): (**a**) Test and (**b**) FEM (with the stress contour).

In Table 2, the numerical achieved load-to-displacement values of the CFRHS columns are compared with the experimental outcomes. It can be seen that the FE results are in good agreement with the test results in terms of the ultimate capacity. The average value of the FEM/Test ratio for the ultimate capacity is 0.985. At the same time, by comparing the failure modes of the specimen S1600-0, it is found that the numerical method can obtain similar failure modes to the test, as shown in Figure 10.

### 3.3. Parametric Analysis

Based on the validated numerical model, a series of parametric analyses are further carried out herein. In the analysis, the influences of different key design variables on the load-bearing capacity of the column are discussed. It needs to be noted that the cross-section of the control specimen is RHS 110 × 150 mm, and only one parameter is changed at a time for each study.

(1) Eccentricity

**Figure 11:**
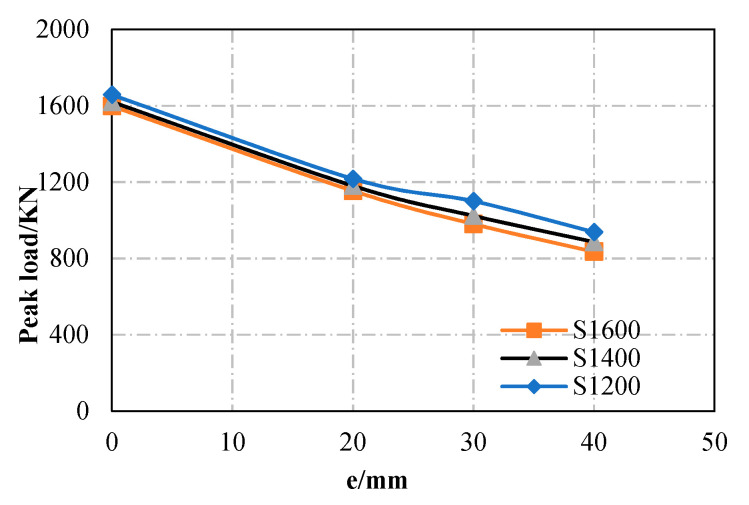
Effect of eccentricity.

It can be seen from the analysis that as the eccentricity (e) increases, the ultimate capacity of the specimens (e.g., S1200, S1400, and S1600) decreases linearly, and the tendency is similar between specimens with varying lengths. When e increases from 0 to 20, 30, and 40 mm, the load-bearing capacity of S1200, S1400, and S1600 increase by 27.2%, 36.4%, and 45.5% on average (see Figure 11). It can be concluded that the eccentricity has a significant impact on specimens of different lengths.

(2) Shell thickness

**Figure 12:**
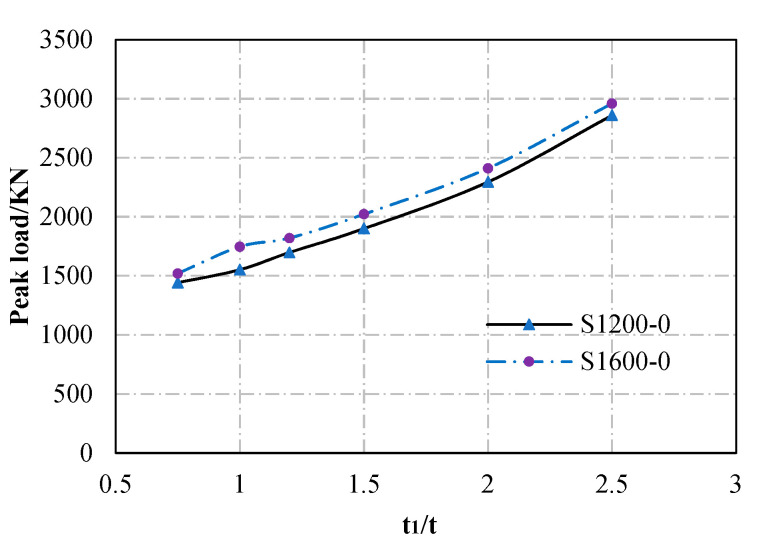
Effect of shell thickness.

Taking the section thickness *t*_1_ = *t*_2_ = 7.82 mm and *t_w_* = 2.92 mm as the control thickness (t), the ultimate capacity of the specimens with different thicknesses is compared in Figure 12. The comparison indicates that the thickness of the steel section also has a considerable effect on the load-bearing capacity of the column. For S1200-0 specimens, when the thickness is increased from 0.7 t to 1.0 t, 1.2 t, 1.5 t, 2.0 t, and 2.5 t, the ultimate capacity of the specimens increases linearly, and the increase percentages are 7.6%, 17.8%, 31.8%, 59.2%, and 98.3%, respectively. The same trend also appears in S1600-0 specimens. This is because with the increase of the shell thickness the critical buckling stress increase in the specimen.

(3) Steel and concrete material properties

**Figure 13:**
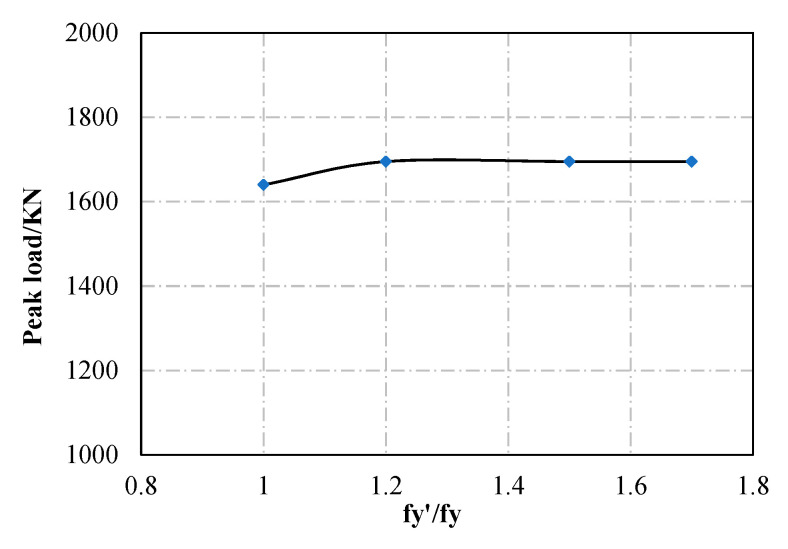
Effect of steel yield strength (S1600).

**Figure 14:**
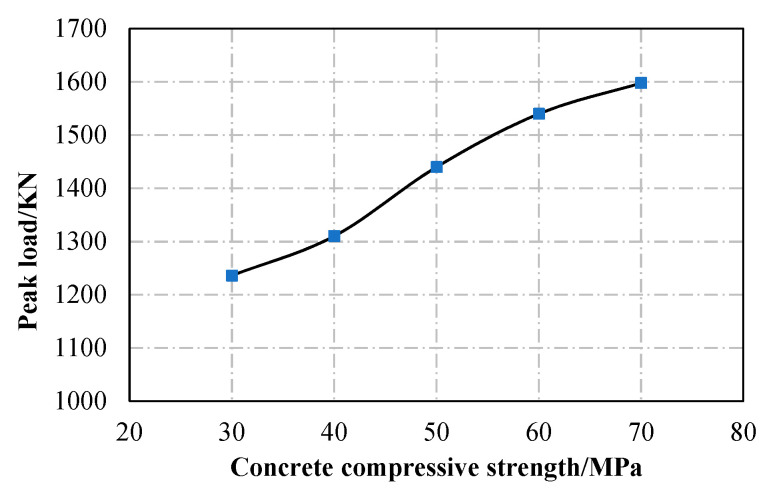
Effect of concrete compressive strength (D1600).

As shown in Figure 13, the yield strength of the steel has a limited influence on the ultimate capacity of the specimen, because the ultimate capacity of the specimen is controlled by the strength of the concrete. When the compressive strength of the concrete increases from C30 to C40, C50, C60, and C70, the ultimate capacity of the specimen increases by 6.0%, 16.5%, 24.6%, and 29.3%, respectively, as shown in Figure 14.

## 4. Analytical Design Approach

In this section, the theoretical design approach for the load-bearing capacity of eccentrically compressed CFRHS columns with unequal flange thickness is proposed. The prediction is conducted based on the following assumptions:

(1) The lateral effect of the steel column is ignored.
(2) It is assumed that the stress–strain curve of the steel and concrete is following ideal elastoplasticity.
(3) The deformations of the steel tube and the core concrete are coordinated.
(4) The deflection of the column is assumed as a sine half-wave under the failure load.

**Figure 15:**
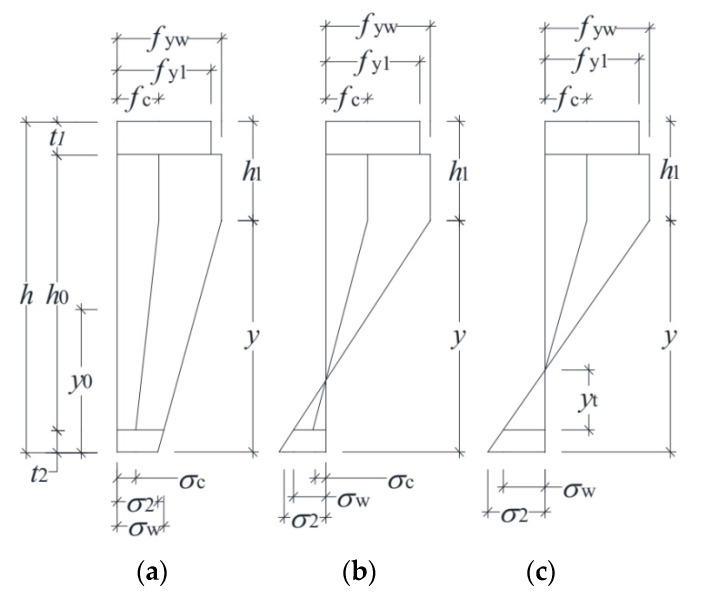
Stress distribution: (**a**) Case 1-1, (**b**) Case 1-2, and (**c**) Case 2.

In the critical state, the concrete in the column develops partial plasticity according to the stress distribution observed in the test, and the depth of the sectional compressive plastic zone is assumed greater than the flange thickness (*h*_1_ > *t*_1_). The behavior of the test specimens can be categorized into two cases, as shown in Figure 15.

The physical centroid position of the unequal walled CFRHS can be calculated by the following formula:

(4) $$y_{0}=\frac{f_{y1}bt_{1}(h-t_{1}/2)+f_{y2}bt_{2}{}^{2}/2+f_{yw}h_{0}b^{\prime }(h-t_{1}-h_{0}/2)}{N_{SC}}$$

where $m=f_{c}/f_{yw}$, $b^{\prime }=2t_{w}+mb_{0}$, $N_{sc}=f_{y1}t_{1}b+f_{y2}t_{2}b+f_{yw}h_{0}b^{\prime }$.

### 4.1. Case Study

#### Case 1

In the critical state, the hogging flange is in the elastic stage, and no crack occurs in the concrete. The flange on the sagging side of the column yields completely, and the web yields partially. Meanwhile, only part of the concrete reaches the compressive strength. Case 1-1 has the same formula format as Case 1-2; the only difference is that, for Case 1-1, the stress variables $\sigma_{2}$, $\sigma_{w}$, and $\sigma_{c}$ are positive, while for Case 1-2, the variables are negative.

The relationship between the curvature and stress of the mid-height section of the column is as follows:

(5) $$\frac{1}{\rho }=y_{m}{\left(\frac{\pi }{l}\right)}^{2}=\frac{f_{yw}-\sigma_{2}}{Ey}=\frac{f_{yw}-\sigma_{w}}{E(y-t_{2})}$$

where $y_{m}$ is the deflection and $E_{s}$ is the Elasticity module. Then we get the following:

(6) $$\begin{array}{l} f_{yw}-\sigma_{2}=E{\left(\pi /l\right)}^{2}yy_{m}\\ f_{yw}-\sigma_{w}=E{\left(\pi /l\right)}^{2}(y-t_{2})y_{m} \end{array}\}$$

The balance condition of the internal and external forces of the section is as follows:

(7) $$N=N_{SC}-(f_{y2}-f_{yw})t_{2}b-\frac{1}{2}(f_{yw}-\sigma_{2})yb+\frac{1}{2}(1-m)(f_{yw}-\sigma_{w})(y-t_{2})b_{0}$$

(8) $$\begin{array}{l} N(e+y_{m})=(f_{y2}-f_{yw})t_{2}b\left(y_{0}-\frac{t_{2}}{2}\right)+\frac{1}{2}(f_{yw}-\sigma_{2})yb\left(y_{0}-\frac{y}{3}\right)\\ -\frac{1}{2}(1-m)(f_{yw}-\sigma_{w})(y-t_{2})b_{0}\left(y_{0}-t_{2}-\frac{y-t_{2}}{3}\right) \end{array}$$

Substitute Equation (6) into Equations (7) and (8):

(9) $$N_{2}=N_{SC}+(f_{y2}-f_{yw})t_{2}b$$

(10) $$M_{2}=(f_{y2}-f_{yw})t_{2}b\left(y_{0}-\frac{t_{2}}{2}\right)$$

(11) $$G_{2}=\frac{1}{2}E{\left(\frac{\pi }{l}\right)}^{2}\left[by^{2}-(1-m)b_{0}{\left(y-t_{2}\right)}^{2}\right]$$

(12) $$H_{2}=\frac{1}{2}E{\left(\frac{\pi }{l}\right)}^{2}\left[by^{2}\left(y_{0}-\frac{y}{3}\right)-(1-m)b_{0}{\left(y-t_{2}\right)}^{2}\left(y_{0}-t_{2}-\frac{y-t_{2}}{3}\right)\right]$$

then

(13) $$N=N_{2}-G_{2}y_{m}=N_{in2-3}$$

(14) $$N(e+y_{m})=M_{2}+H_{2}y_{m}=M_{in2-3}$$

In this case, we get the following:

(15) $$G_{2}^{\prime }=E{\left(\frac{\pi }{l}\right)}^{2}\left[b^{\prime }y+(1-m)b_{0}t_{2}\right]$$

(16) $$H_{2}^{\prime }=\frac{1}{2}E{\left(\frac{\pi }{l}\right)}^{2}\left[by(2y_{0}-y)-(1-m)b_{0}(y-t_{2})(2y_{0}-y-t_{2})\right]$$

and *y* can be achieved as follows:

(17) $$y=\frac{H_{2}^{\prime }(N_{2}-N)}{E{\left(\pi /l\right)}^{2}b^{\prime }(eN-M_{2})}-\frac{(1-m)b_{0}t_{2}}{b^{\prime }}$$

The boundary condition for this case is as follows:

$$t_{2}\leq y\leq h-t_{1},\left|\sigma_{2}\right|\leq f_{y2},\;\left|\sigma_{w}\right|\leq \min\left\{f_{y2},f_{yw}\right\},\;-f_{c}\leq \frac{\sigma_{w}}{m}\leq f_{ct}$$

#### Case 2

In this case, the stress distribution under the critical stage is the same as Case 1, but the concrete cracks in the tension zone are assumed out-of-work. The balance equation can be derived based on the Case 1, but the concrete in the tension zone need to be eliminated in the equation. According to Equation (6), the tensile stress $\sigma_{w}$ can be calculated as follows:

(18) $$\sigma_{w}=E{\left(\pi /l\right)}^{2}y_{m}(y-t_{2})-f_{yw}$$

According to the relationship between the curvature and stress, the concrete depth in the compression zone can be obtained as follows:

(19) $$y_{t}=\frac{\sigma_{w}}{E{\left(\pi /l\right)}^{2}y_{m}}$$

The balance equation includes the internal force and internal moment of the concrete:

(20) $$\Delta N_{2-4}=\frac{1}{2}b_{0}m\sigma_{w}y_{t},\;\Delta M_{2-4}=-\Delta N_{2-4}\left(y_{0}-t_{2}-\frac{y_{t}}{3}\right)$$

Substitute Equations (18) and (19) into Equation (20), to obtain the following:

(21) $$\Delta N_{2-4}=\frac{mb_{0}}{2E{\left(\pi /l\right)}^{2}y_{m}}{\left[E{\left(\pi /l\right)}^{2}y_{m}(y-t_{2})-f_{yw}\right]}^{2}$$

(22) $$\Delta M_{2-4}=-\Delta N_{2-4}\left\{y_{0}-t_{2}-\frac{1}{3}\left[y-t_{2}-\frac{f_{yw}}{E{\left(\pi /l\right)}^{2}y_{m}}\right]\right\}$$

According to the above expression, the balanced equation of this case can be obtained as follows:

(23) $$\begin{array}{r} N=N_{in2-3}+\Delta N_{2-4}\\ N(e+y_{m})=M_{in2-3}+\Delta M_{2-4} \end{array}\}$$

The boundary condition is as follows:

$$h_{1}>t_{1},\;\sigma_{2}\leq f_{y2},\;\sigma_{w}\leq f_{yw},\;\sigma_{c}>f_{ct}$$

### 4.2. Results Comparison

The ultimate capacity of each specimen calculated by the proposed theoretical formulas is listed in Table 3. It can be seen that the design approach from AISC 360-10 (*N_AISC_*) [25] is conservative in prediction the load-bearing capacity of the CFRHS columns with non-equal wall thickness, with the average ratio (*N_AISC_*/*N_Test_*) is 1.116. The analytically predicted value (*N_Analytical_*) has a closer correlation with the test results (*N_Test_*), with the average ratio (*N_Analytical_*/*N_Test_*) is 0.966, and the mean square error is 0.017.

It indicates from the table that as slenderness increases, the load-bearing capacity of the eccentrically compressed unequal-walled CFRHS columns decreases. Under the same slenderness, the load-bearing capacity of the columns increases with increasing *t*_2_/*t*_1_ (ratio between top and bottom flange thicknesses), which is due to that the eccentricity decreases as increasing *t*_2_/*t*_1_. Therefore, the flange thickness ratio (*t*_2_/*t*_1_) can be changed intentionally by designers to reduce the eccentricity of the column.

## 5. Conclusions

In this paper, the compression performance of the simply support CFRHS columns was investigated based on experimental and numerical approaches. An analytical method was further presented, to predict the ultimate capacity of the column. The following conclusions can be drawn from the study:

(1) The failure mode of the axial compression column is the local buckling of the tube on the top side, and the final failure mode of the eccentric compressed column is the buckling on the middle section of the column. For the equal-walled and unequal-walled CFRHS columns, when the thickness of the flange and web is relatively small, the bucking is tending to occur on these regions; hence, the outer steel tube needs to be reinforced to resist local buckling.
(2) It is found that axially compressed unequal-wall-thickness CFRHS columns have good ductility when compared with the unequal-wall-thickness columns.
(3) It is indicated that, with the same section area, slenderness, and eccentricity, the load-bearing capacity of eccentrically compressed CFRHS column with unequal wall thickness can improve 20% more than columns with equal shell thickness. Therefore, in order to improve the load-bearing capacity, we recommend designing the eccentric compressed column with unequal-wall-thickness section.
(4) The numerical simulation method of the compressed CFRHS column is in good agreement with the experimental results, which verifies the correctness of the finite element simulation method. Parametric studies show that the eccentricity, shell thickness, and concrete strength have a significant impact on the load-bearing capacity of the column.

## Figures and Tables

**Table 1:** List of specimen parameters.

Specimen	*b*/mm	*h*/mm	*L*/mm	λ	*t*_1_/mm	*t*_2_/mm	*t*_w_/mm	*e*/mm	*f_y_*_1_/MPa	*f_y_*_2_/MPa	*f_y_*_w_/MPa	*f_cu_*/MPa
S1200-0	110	150	1200	27.7	7.82	7.82	2.92	0	267.9	267.9	340.7	69.6
S1200-20	110	150	1200	27.7	7.82	7.82	2.92	20	267.9	267.9	340.7	69.6
S1200-40	110	150	1200	27.7	7.82	7.82	2.92	40	267.9	267.9	340.7	69.6
D1200-0	110	150	1200	27.7	5.88	9.68	2.92	0	294.4	263.2	340.7	69.6
D1200-20	110	150	1200	27.7	5.88	9.68	2.92	20	294.4	263.2	340.7	69.6
D1200-40	110	150	1200	27.7	5.88	9.68	2.92	40	294.4	263.2	340.7	69.6
S1600-0	110	150	1600	37.0	7.82	7.82	2.92	0	267.9	267.9	340.7	69.6
S1600-20	110	150	1600	37.0	7.82	7.82	2.92	20	267.9	267.9	340.7	69.6
S1600-40	110	150	1600	37.0	7.82	7.82	2.92	40	267.9	267.9	340.7	69.6
D1600-0	110	150	1600	37.0	5.88	9.68	2.92	0	294.4	263.2	340.7	69.6
D1600-20	110	150	1600	37.0	5.88	9.68	2.92	20	294.4	263.2	340.7	69.6
D1600-40	110	150	1600	37.0	5.88	9.68	2.92	40	294.4	263.2	340.7	69.6

**Table 2:** Comparison between the finite element (FE) and test results.

Specimen	*N*_ue_/kN	*N*_u-FEM_/kN	*N*_u-FEM_/*N*_ue_
S1200-0	1655	1657	1.001
S1200-20	1215.5	1217	1.001
S1200-40	939.5	938	0.998
D1200-0	1610	1600	0.994
D1200-20	1223	1205	0.985
D1200-40	995	896	0.901
S1600-0	1660.5	1636	0.985
S1600-20	1187.5	1154	0.972
S1600-40	936.5	835	0.892
D1600-0	1580	1602	1.014
D1600-20	1196.5	1260	1.053
D1600-40	953.5	978	1.026
Mean			0.985
SD			0.047

**Table 3:** Results comparison.

Specimen	*t*_2_/*t*_1_	*λ*	*N_Test_*/kN	*N*_Analytical_/kN	*N_AISC_*/kN	*N*_Analytical_/*N_Test_*	*N_AISC_*/*N_Test_*	Case Type
S1200-20	1.00	27.7	1215.5	1172	1315	0.964	1.082	1
S1200-40	1.00	27.7	939.5	933	1060	0.993	1.128	2
D1200-20	1.65	27.7	1223	1208	1335	0.988	1.092	1
D1200-40	1.65	27.7	995	954	1120	0.959	1.126	2
S1600-20	1.00	37	1187.5	1128	1304	0.950	1.098	1
S1600-40	1.00	37	936.5	889	1040	0.949	1.111	2
D1600-20	1.65	37	1196.5	1166	1325	0.975	1.107	1
D1600-40	1.65	37	953.5	909	1130	0.953	1.185	2
Mean						0.966	1.116	
SD						0.017	0.032	
